# CT Texture analysis and CT scores for characterization of fluid collections

**DOI:** 10.1186/s12880-021-00718-w

**Published:** 2021-12-06

**Authors:** Hans-Jonas Meyer, Benedikt Schnarkowski, Jakob Leonhardi, Matthias Mehdorn, Sebastian Ebel, Holger Goessmann, Timm Denecke

**Affiliations:** 1 Department of Diagnostic and Interventional Radiology, University of Leipzig, Liebigstraße 20, 04103 Leipzig, Germany; 2 Department of Visceral, Transplant, Thoracic and Vascular Surgery, University of Leipzig, Leipzig, Germany

**Keywords:** Texture analysis, CT, Fluid collection, Drainage treatment

## Abstract

**Background:**

Texture analysis derived from Computed tomography (CT) might be able to better characterize fluid collections undergoing CT-guided percutaneous drainage treatment. The present study tested, whether texture analysis can reflect microbiology results in fluid collections suspicious for septic focus.

**Methods:**

Overall, 320 patients with 402 fluid collections were included into this retrospective study. All fluid collections underwent CT-guided drainage treatment and were microbiologically evaluated. Clinically, serologically parameters and conventional imaging findings as well as textures features were included into the analysis. A new CT score was calculated based upon imaging features alone. Established CT scores were used as a reference standard.

**Results:**

The present score achieved a sensitivity of 0.78, a specificity of 0.69, area under curve (AUC 0.82). The present score and the score by Gnannt et al. (AUC 0.81) were both statistically better than the score by Radosa et al. (AUC 0.75). Several texture features were statistically significant between infected fluid collections and sterile fluid collections, but these features were not significantly better compared with conventional imaging findings.

**Conclusions:**

Texture analysis is not superior to conventional imaging findings for characterizing fluid collections. A novel score was calculated based upon imaging parameters alone with similar diagnostic accuracy compared to established scores using imaging and clinical features.

## Background

Fluid collections are common and can represent ascites, seroma, hematoma, biloma, lymphocele, or abscess depending on the localization and underlying cause [1–4]. The mortality rates associated with infected fluid collections range from 4% in treated cases to more than 80% in untreated cases [4]. Hence, early and correct diagnosis and treatment are crucial to determine the clinical outcome [4, 5]. Many of these patients are suitable for Computed tomography (CT)-guided percutaneous drainage due to advances in CT-guided treatment, whereas surgery is only performed in few selected cases [5].

The important aspects to diagnose an infected fluid collection is clinical investigation, serological parameters, of most importance the inflammation parameter C-reactive protein (CRP) and lastly imaging modalities. CT is commonly performed in these patients to detect possible septic foci and to plan the following treatment.

Some imaging features were reported indicative of an infection of a fluid collection, including air entrapment, rim contrast media enhancement and Hounsfield unit (HU) attenuation. However, these imaging features by itself cannot reliably exclude infection due to lack of a sufficient accuracy for correct diagnosis [3, 4].

Those imaging and clinical features lead to a proposed score by Gnannt et al., which utilized CT features, clinical and serological parameters to predict the likelihood of infected abdominal fluid collections undergoing CT-guided drainage treatment in postsurgical patients [6]. In this study, the score achieved an excellent area under the curve of 0.96 in a validation cohort comprising 30 patients [6].

To this date, only one other study performed a validation analysis for this score and proposed a more accessible combination of imaging findings with only one clinical parameter, namely CRP to correctly diagnose infected fluid collections [4].

This proposed simplified score achieved an even higher accuracy than the first one by Gnannt et al. with a reported sensitivity of 93% and a specificity of 80% [4].

Texture analysis is a novel imaging analysis, which quantifies radiological images and can thereby provide imaging biomarkers [7–9]. Most commonly are images derived from Computed tomography (CT) due to its robust imaging acquisition and high availability in clinical routine [7–9]. In recent years, texture analysis was predominantly investigated in oncological imaging to better characterize tumor behavior [9]. Thus, it is acknowledged that texture analysis can give deeper insight into the microstructure of tissues. Presumably, texture analysis might also help to better characterize different components of fluid collections to discriminate hematoma and bacterial debris. However, texture analysis has not been employed for this purpose yet.

The principal hypothesis for this present work is that texture analysis might be able to reflect distinctive differences of fluid collections and might be a reliable tool to better characterize fluid collections and the need of drainage treatment. As a second part, the study performed a validation analysis for the two proposed scores to test the external validity and to validate, whether these scores can also be used in non-surgical patients and fluid collections of the pleura.

## Methods

The radiological database of a university hospital was retrospectively screened for patients treated with CT-guided percutaneous drainage between January 2017 and December 2020.

Inclusion criteria were sufficient contrast enhanced CT for septic evaluation within 24 h before the intervention, sufficient native planning CT, available serological and microbiological evaluation. Overall, 738 patients were identified in the database, who were treated with a CT-guided percutaneous drainage. Several patients had to be excluded for the following reasons: n = 252 patients with no contrast enhanced CT within 48 h before drainage, n = 88 no microbiology, n = 56 with no sufficient clinical data for the score calculation, n = 18 no serological data, n = 4 CT texture analysis not possible due to artifacts.

**Fig. 1:**
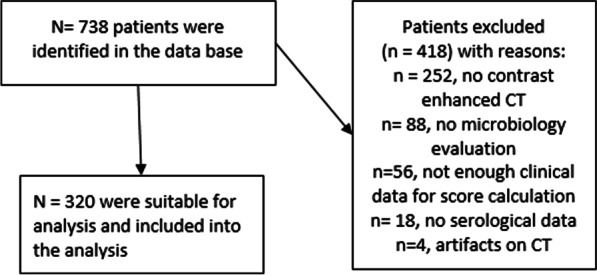
Flow chart of the acquisition of the patient sample. After exclusion for the given reasons, the final patient sample 320 patients with overall 402 fluid collections were included into the present study

Finally, 320 patients (n = 119 females, 35.6%, mean age of 62 ± 14 years, range 20–94 years) were included into the analysis. Figure 1 gives an overview of the patient acquisition.

### Image analysis

At first, the CT images were qualitatively evaluated by one reader using the picture archiving and communication system (Syngo Plaza, Siemens). In unclear cases, a second reader with 5 years of experience in general radiology was consulted, and a consensus was met.

The quantitative evaluation of the CT images was performed accordingly to Gnannt et al. [6].

- The maximum length was measured.
- Hounsfield units (HU) measured with a region of interest (ROI) within the fluid collection
- Presence or absence of a contrast enhancing rim of the fluid collection.
- Presence or absence of fat stranding surrounding the fluid collection.
- Presence or absence of entrapped gas within the collection.

### Clinical data acquisition

The electronic medical records system was utilized to extract the following clinical data: age, sex, underlying disease, presence of medication with immunosuppressive drugs (e.g. glucocorticoids or cytostatics), medication with antibiotics, body temperature and blood sample including CRP, white blood cell counts, procalcitonin and interleukin 6 within 6 h before percutaneous drainage.

### Image acquisition

CT imaging was performed in a clinical setting with a 128 or 256 slices CT scanner (Ingenuity or iCT, Philips, Amsterdam, Netherlands). In all cases, 90 mL of iodinated intravenous contrast medium was given at a rate of 1.5 to 3.5 mL/s by a power injector (Medtron GmbH, Germany), with a scan delay of 70 s after onset of injection for clinical evaluation of septic foci. Typical imaging parameters were 120 kVp, 150 to 300 mAs, and a slice thickness of 1 mm.

The CT intervention was performed with a 16 slices scanner (Big Bore 16, Philips, Amsterdam, Netherlands). In all patients, a native CT spiral scan was performed for planning of the intervention covering the body area of the known fluid collection. Typical imaging parameters were 120 kVp, 150 mAs, and a slice thickness of 2 mm.

In all cases, a percutaneous drainage was placed within the fluid collection, size ranging from 8-F to 22-F depending on clinical presenting and localization.

### Score calculation

The previously proposed score by Gnannt et al. was calculated according to the publication [6]. In short, 10 possible points can be obtained. Clinically parameters are known or absent Diabetes, CRP over or under 100 mg/L, gas entrapment, CT attenuation under 10 HU, between 10 and 20 HU, over 20 HU. A score of 0–2 points was defined for a low probability of infection, 3–10 for a high probability of infection.

The second score by Radosa et al. was also calculated [4]. This score uses only CRP as a serological parameter 0 or 4 points with a cut-off value (150 mg/L), CT attenuation under or over 20 HU 0 or 2 points, gas entrapment 0 or 3 points, wall enhancement 0 or 2 points. The proposed cut-off value is 5, under for low probability of infection, over for high probability of infection.

### Texture analysis

One the next step, the texture analysis was performed blinded to the microbiological results. CT images further processed with the free available texture analysis software MaZda (version 4.7, available at http://www.eletel.p.lodz.pl/mazda/) [10, 11]. Images were analyzed in 1 mm soft tissue kernel reconstructed slices. A polygonal ROI was placed on the largest, representative slide of the fluid collection. The ROI was clearly drawn within the boundary of the fluid collection. Moreover, entrapped gas was avoided in the ROI, which could influence the texture analysis results. For each ROI, gray-level normalization was performed, using the limitation of dynamics to μ ± 3 SD (μ gray level mean, SD standard deviation) to minimize the influence of contrast and brightness variation, as it was performed previously [12, 13]. The extracted texture features are as follows: gray-level histogram (mean, variance, skewness, kurtosis, percentiles (1, 10, 50, 90, 99%), co-occurrence matrix (angular second moment, contrast, correlation, entropy, sum entropy, sum of squares, sum average, sum variance, inverse difference moment, difference entropy, difference variance (for four directions and five interpixel distances (offsets; n = 1 to 5)), run-length matrix (run-length non-uniformity, gray-level non-uniformity, long run emphasis, short run emphasis, fraction of image in runs)), absolute gradient (gradient mean, variance, skewness, kurtosis, non-zeros), autoregressive model (theta 1 to 4, sigma), and wavelet transform (energies of wavelet transform coefficients in sub-bands LL, LH, HL, HH). Altogether, 279 texture features were retrieved from every fluid collection.

**Fig. 2:**
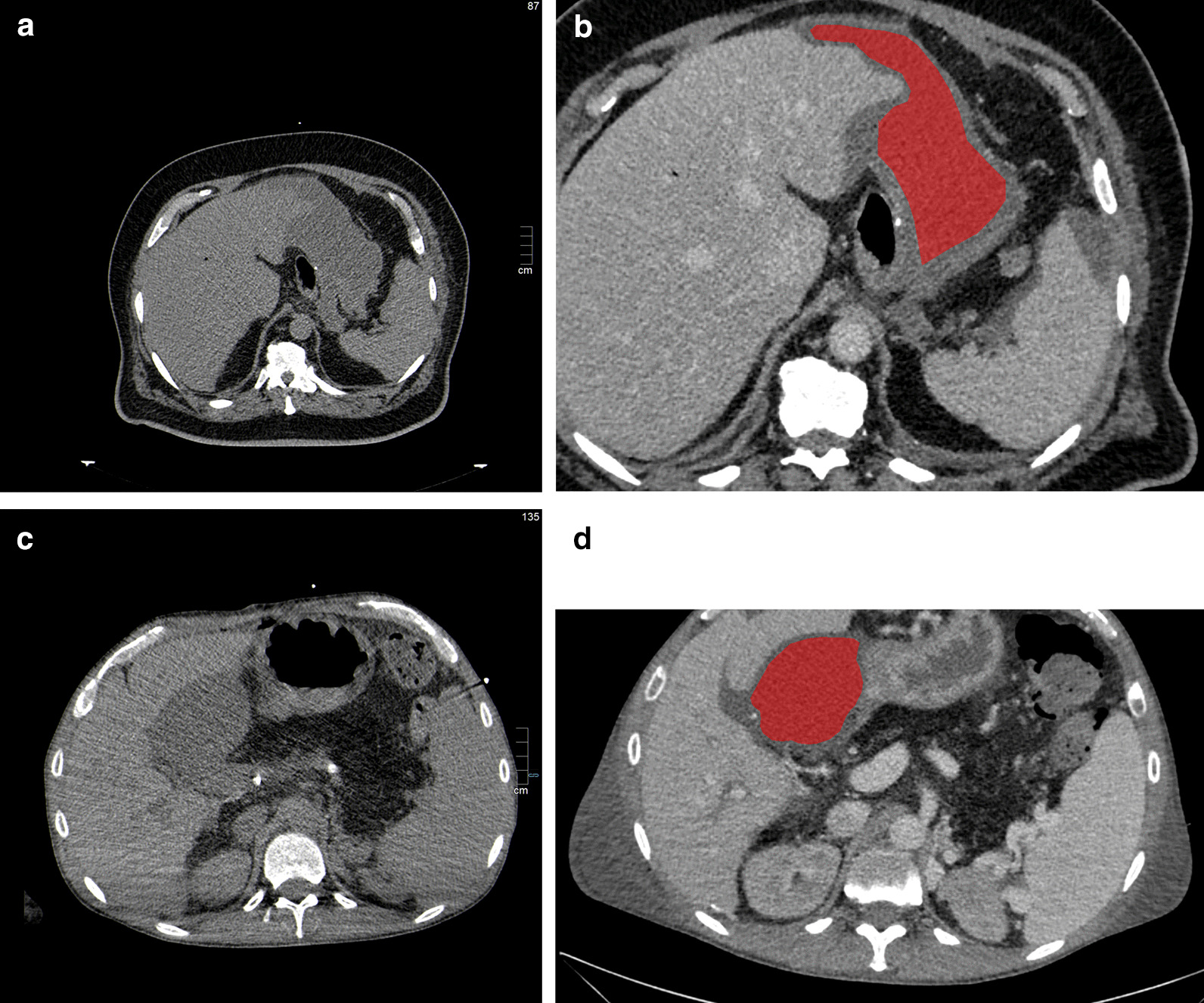
**a** Representative 66-years old male case of the patient sample with an infected perigastric fluid collection after sleeve-gastrectomy surgery 5 days ago. A rim contrast media enhancement and higher Hounsfield units above 20 can be appreciated. There are no gas entrapments or perifocal stranding. Small free perisplenic free fluid can also be seen. **b** The drawn region of interest of the fluid collection. **c** Representative 54-years old male case with a non-infected fluid collection. The fluid collection is located at the hilum of the liver after liver transplantation 4 days ago. No contrast media enhancement, gas entrapment, perifocal stranding or higher Hounsfield units can be appreciated. **d** The drawn region of interest of the fluid collection

Figure 2 displays 2 representative cases of the patient sample, one patient with an infected fluid collection and one with a sterile fluid collection.

### Microbiological analysis

All fluid collections were microbiologically analyzed during clinical work up blinded to the imaging results. As part of the CT-guided intervention, at least 5 ml of drained fluid was sent for microbiological analysis. According to current recommendations, fluid collections were considered infected if leukocytes and bacteria were detected on the Gram stain and/or the culture was positive for bacteria or fungi [6]. If these were negative, the fluid collection was classified as non-infected.

### Statistical analysis

Statistical analysis was performed using GraphPad Prism 5 (GraphPad Software, La Jolla, CA, USA) and SPSS STATISTICS (IBM, Version 25.0; Armonk, NY, USA). Collected data were evaluated by means of descriptive statistics (absolute and relative frequencies). Spearman's correlation coefficient (r) was used to analyze associations between texture features and the proposed scores. Differences of the texture features between infected and non-infected fluid collections were investigated by two tailed Mann–Whitney test. Then, a Receiver operating characteristics (ROC) analysis was performed and compared between texture features and the scores. AUC characteristics between the scores were compared with DeLong test. A Random-Forest classifier as well as a multivariate analysis was used to construct a predictive model to classify infected fluid collections using qualitative as well as quantitative imaging parameters and serological parameters. In all instances, *p* values < 0.05 were taken to indicate statistical significance.

## Results

### Clinical and radiological parameters

An overview of the descriptive statistics of the investigated demographic, serologic and imaging parameters is given by Table 1.

**Table 1:** Overview of the demographic, serologic and imaging parameters of the patient sample

Parameter	Non-infected fluid collections (n = 147)	Infected fluid collections (n = 255)	*p* value
Age, years	62 ± 15 (21–94)	62 ± 14 (20–90)	0.9
Sex, male/female	70/37	136/77	0.4
Diabetes	26 (21.5%)	63 (29.6%)	0.07
Immunosuppressive drugs	16 (13.2%)	31 (14.6%)	0.4
Antibiotics	92 (76%)	172 (80.8%)	0.2
C-reactive protein, mg/L	152 ± 101 (1–444)	177 ± 102 (1–435)	0.02
Leukocytes, 10^9^/L	13.4 ± 6.9 (0.7–38.5)	15.3 ± 8 (2.3–81.2)	0.009
Procalcitonin, ng/ml	3.2 ± 8.1 (0.1–41.4)	6 ± 15.6 (0.1–108.5)	0.02
Interleukin-6, pg/ml	431 ± 701 (38–1850)	3061 ± 6953 (38–28,687)	0.4
Attenuation, HU	13 ± 11 (0–52)	18 ± 8 (0–58)	< 0.001
Maximum diameter, cm	8.5 ± 3.9 (2.2–25.5)	7.8 ± 3.4 (2.1–20)	0.07
Wall enhancement	59 (40.1%)	153 (60%)	< 0.001
Fat stranding	57 (38.8%)	161 (63.1%)	< 0.001
Entrapped gas	30 (20.4%)	148 (58%)	< 0.001

Data are expressed as mean ± standard deviation (range) or frequencies with percentages

*p* values are calculated with Mann–Whitney Test or Fisher’s exact test where appropriate

HU = Hounsfield Units

Overall, 402 fluid collections were investigated. In most patients one fluid collection was identified, in 68 cases 2 fluid collections were identified and in 7 cases 3 fluid collections were identified. There were 147 non-infected (36.7%) and 255 infected fluid collections (63.3%). In all patients, a successful percutaneous CT-guided drainage was placed within the fluid collection and the fluid was microbiologically evaluated.

Table 2 gives an overview of the underlying diseases of the patients and localizations of the fluid collections. In 52.2% of all patients, a malignant tumor was known, 30% of all patients suffered from a septic disease.

**Table 2:** Overview of the underlying diseases and localization of the fluid collection

Underlying disease	Patients
Malignancy	167 (52.2%)
Infection	96 (30%)
Trauma	8 (2.5%)
Vascular	12 (3.8%)
Others	37 (11.5%)
*Localization*	
Intra- and extraperitoneal cavity	112 (35%)
Pleural	89 (27.8%)
Liver	63 (19.7%)
Pelvic	39 (12.2%)
Others	17 (5.3%)

### Diagnostic accuracy of the investigated scores

The resulting score was only defined by imaging parameters (Table 3) in the multilinear regression: No serological or clinical parameters were utilized by this analysis.

**Table 3:** The new proposed score identified by multivariate regression analysis

Present score	β (regression coefficient)	Points
*Fat stranding*		
No	0	0
Yes	1.6	2
*Gas entrapment*		
No	0	0
Yes	3	3
*CT attenuation, HU*		
≤ 10	0	0
> 10	4	4
Minimum total score		0
Maximum total score		9
Cut-off ≥ 5 points		

HU = Hounsfield units

The accuracy of the investigated scores is shown by Table 4. The present score achieved a sensitivity of 0.78, a specificity of 0.69 with an AUC 0.82.

**Table 4:** Comparison between the analyzed scores in regard of diagnostic accuracy

	AUC	95% CI
Gnannt score	0.81	0.77 to 0.85
Radosa score	0.75	0.70 to 0.79
Present score	0.82	0.78 to 0.86

AUC = area under curve, CI = Confidence interval

**Fig. 3:**
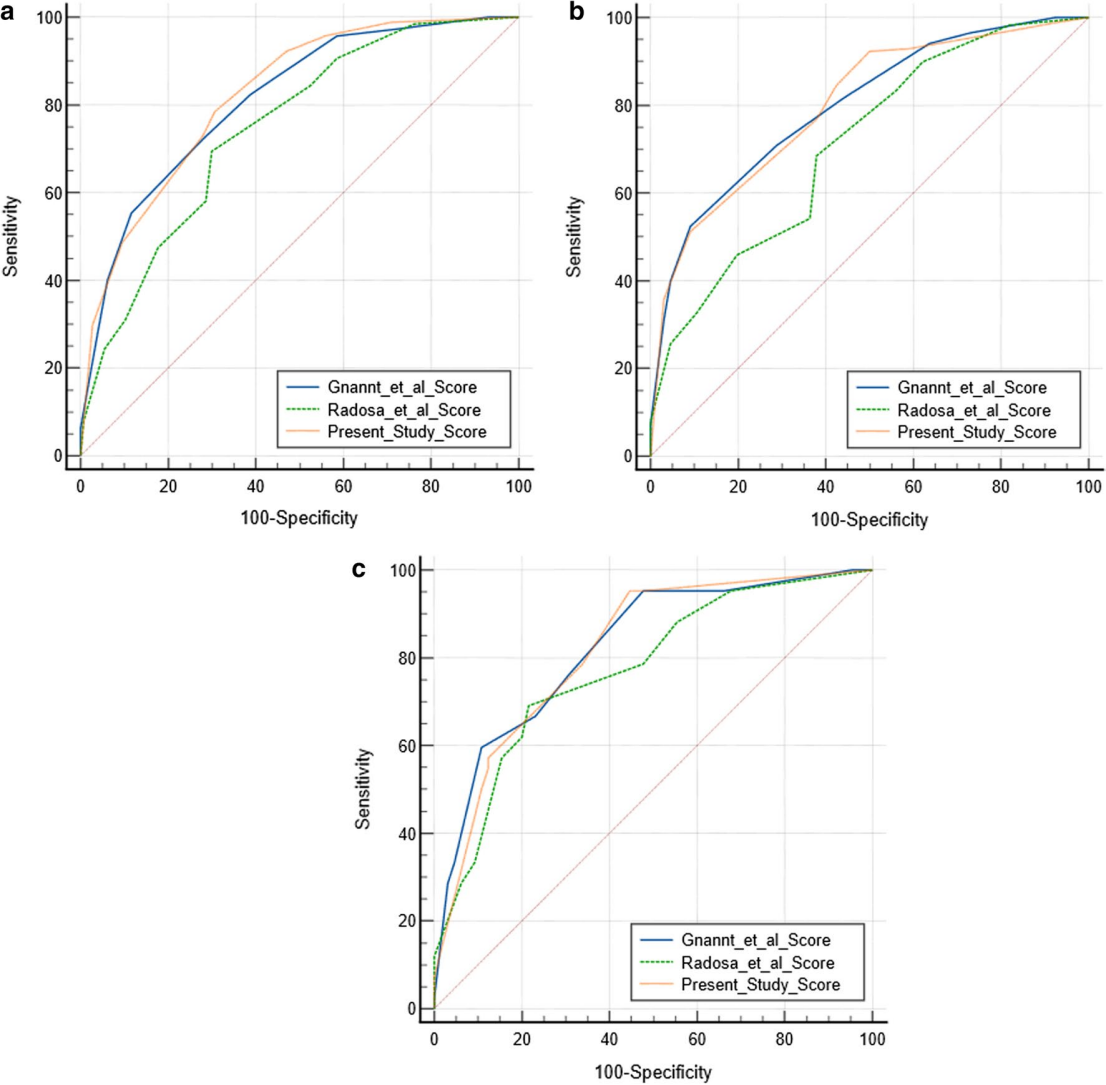
**a** ROC curves of the investigated CT scores in the overall patient sample. Gnannt et al. achieved an AUC of 0.81, Radosa et al. an AUC of 0.75 and the present score an AUC of 0.82. The present score and the score by Gnannt et al. were both significantly better than the score by Radosa et al. **b** ROC curve of the investigated CT score in the subanalysis of only postoperative patients. Gnannt et al. achieved an AUC of 0.80, Radosa et al. an AUC of 0.71 and the present score an AUC of 0.80. The present score and the score by Gnannt et al. were both significantly better than the score by Radosa et al. **c** ROC curve of the investigated CT score in the subanalysis of pleural fluid collections. Gnannt et al. achieved an AUC of 0.82, Radosa et al. an AUC of 0.77 and the present score an AUC of 0.82

Gnannt et al. achieved an AUC of 0.81 (95% CI 0.77–0.85), Radosa et al. an AUC of 0.75 (95% CI 0.70–0.79) and the present score an AUC of 0.82 (95% CI 0.78–0.86) (Fig. 3a). The present score and the score by Gnannt et al. were both significantly better than the score by Radosa et al.

The present score was significantly superior to the score by Radosa et al. (*p* = 0.04), and not inferior to the score by Gnannt et al. (*p* = 0.23). The score by Gnannt et al. was significantly better than the score by Radosa et al. (*p* = 0.002). Figure 3a shows the corresponding ROC graphs.

There was a strong positive correlation between the score by Gnannt et al. with the score by Radosa et al. (r = 0.63, *p* < 0.001). The present score correlated both with Gnannt et al. (r = 0.80, *p* < 0.001) and the score by Radosa et al. (r = 0.70, *p* < 0.001).

### Diagnostic accuracy for postoperative and pleural fluid collection

Subanalyses were performed to further assess the diagnostic accuracy of the scores.

Overall, 234 fluid collections were included into the analysis only investigating postoperative fluid collections.

The AUC values did not change significantly: Gnannt et al. achieved an AUC of 0.80 (95% CI 0.74–0.85), Radosa et al. an AUC of 0.71 (95% CI 0.65–0.77) and the present score an AUC of 0.80 (95% CI 0.74–0.85) (Fig. 3b). The present score and the score by Gnannt et al. were both significantly better than the score by Radosa et al.

Overall, 107 fluid collections were included into the analysis of pleural fluid collections.

Again, the AUC values did not change significantly: Gnannt et al. achieved an AUC of 0.82 (95% CI 0.74–0.89), Radosa et al. an AUC of 0.77 (95% CI 0.68–0.85) and the present score an AUC of 0.82 (95% CI 0.73–0.89) (Fig. 3c).

### Accuracy of imaging and clinical findings

Table 5 gives an overview of the accuracy to predict infected fluid collections of single imaging and clinical features. CRP was the best clinical feature achieving a sensitivity of 0.76 and a specificity of 0.40 employing a threshold value of 114 mg/dl. The best imaging feature was HU, which achieved a sensitivity of 0.87 and a specificity of 0.55 employing a threshold value of 11 HU.

**Table 5:** Comparison of the diagnostic accuracy of the clinical and imaging features

Parameter	Threshold-value	Sensitivity	Specificity	Youden-Index
BMI	28.2 kg/mm^2^	0.30	0.80	0.09
Leucocytes	14.8 10^9^/L	0.49	0.67	0.16
CRP	114 mg/L	0.76	0.40	0.16
Fat stranding	Positive	0.63	0.61	0.24
Air entrapment	Positive	0.58	0.80	0.38
Enhancement	Positive	0.60	0.60	0.20
HU	10.5	0.87	0.55	0.42

HU = Hounsfield Units, BMI = Body mass index, CRP = C-reactive protein

### Texture analysis parameters

Several texture features were significantly different between infected and non-infected fluid collections. Table 6 gives an overview of the statistically significant features. However, no texture features achieved a better AUC than the conventional imaging features. Therefore, no texture feature was included into the proposed score.

**Table 6:** Overview of the statistically significant texture parameters between infected and non-infected fluid collections derived from the contrast enhanced CT

Texture feature	*p* value
Mean	< 0.001
Kurtosis	0.016
Perc,01%	< 0.001
Perc,10%	< 0.001
Perc,50%	< 0.001
Perc,90%	< 0.001
Perc,99%	0.017
_Area_S(1,0)	< 0.001
_Area_S(0,1)	< 0.001
_Area_S(1,1)	< 0.001
_Area_S(1,-1)	< 0.001
S(1,-1) InvDfMom	0.011
_Area_S(2,0)	< 0.001
S(2,0) InvDfMom	0.045
_Area_S(0,2)	< 0.001
_Area_S(2,2)	< 0.001
_Area_S(2,-2)	< 0.001
S(2,-2) InvDfMom	0.011
_Area_S(3,0)	< 0.001
_Area_S(0,3)	< 0.001
_Area_S(3,3)	< 0.001
_Area_S(3,-3)	< 0.001
S(3,-3) InvDfMom	0.026
_Area_S(4,0)	< 0.001
S(4,0) InvDfMom	0.045
_Area_S(0,4)	< 0.001
S(0,4)Entropy	0.046
_Area_S(4,4)	< 0.001
_Area_S(4,-4)	< 0.001
_Area_S(5,0)	< 0.001
_Area_S(0,5)	< 0.001
S(0,5)Entropy	0.046
_Area_S(5,5)	< 0.001
S(5,5)Entropy	0.045
_Area_S(5,-5)	< 0.001
S(5,-5)Entropy	0.037
Horzl_RLNonU ni	< 0.001
Horzl_GLevNon U	< 0.001
Horzl_LngREm ph	0.006
Horzl_ShrtREm p	0.034
Horzl_Fraction	0.015
Vertl_RLNonUn i	< 0.001
Vertl_GLevNon U	< 0.001
Vertl_LngREmp h	0.040
45dgr_RLNonU ni	< 0.001
45dgr_GLevNo nU	< 0.001
45dgr_LngREm ph	0.044
135dr_RLNonU ni	< 0.001
135dr_GLevNo nU	< 0.001
135dr_LngREm ph	0.003
135dr_ShrtRE mp	0.017
135dr_Fraction	0.008
_AreaGr	< 0.001
GrSkewness	0.030
GrKurtosis	0.003
GrNonZeros	0.030
_AreaARM	< 0.001
Sigma	0.039
WavEnHH_s-2	0.048
WavEnHL_s-4	0.022
WavEnHH_s-5	0.034
WavEnHH_s-7	0.011

## Discussion

The present study elucidated whether CT scores can reliably predict infection of a fluid collection undergoing percutaneous CT drainage. A novel score was provided only utilizing CT findings, which was comparably good in regard of accuracy to the published scores. Another finding was that texture analysis derived from CT images does not provide additional value to diagnose infected fluid collections.

Previously, several CT findings were identified to be indicative of infected fluid collection comprising increased attenuation, encapsulation with or without wall enhancement, presence of gas entrapment, and stranding of the surrounding tissue [3, 4, 6]. However, it is also widely acknowledged that these findings considered separately cannot reliably predict the infection of a fluid collection. This was previously shown in a study on 92 patients with postoperative fluid collections achieving an average sensitivity of 83.4% and a specificity of 39.3% utilizing the features gas entrapment and high attenuation fluid (20 or greater HU) [3].

That is why the previously proposed 2 CT scores are based upon a set of imaging features to achieve a better accuracy to diagnose infected fluid collections [4, 6]. Moreover, with combination of imaging and clinical parameters the diagnostic accuracy can be improved.

One rationale of the present study was that texture analysis could better characterize fluid collections due to its ability to quantify heterogeneities of radiological images [7].

This has been extensively shown for oncologic imaging with papers elucidating the possible use of texture analysis to reflect histopathology and microstructure of tumors [7–9]. However, the present analysis can state that texture analysis of CT images is not superior to discriminate infected of non-infected fluid collections compared to conventional imaging findings.

It is widely acknowledged that higher attenuation of a fluid collection is mainly caused by debris [2, 6]. Hemorrhage is another reason, which can result in a higher attenuation in non-infected fluid collections. Yet, higher attenuation over 10 Hounsfield units is the strongest predictor of infected fluid collections, with a good sensitivity and specificity, which is almost as good as the combined scores.

Presumably, debris and other associated findings of an abscess or infected fluid collection do not cause distinctive alterations of texture features in comparison to sterile hematomas and seromas, which can explain the negative results.

Another part of the study was the validation of the two proposed CT scores by Gnannt et al. and Radosa et al. [4, 6]. These scores utilized the conventional imaging findings of an infected fluid collection and CRP as a serological parameter. Gnannt et al. also used the anamnestic feature of a known diabetes, which was not needed by Radosa et al.

Contrary to the previous studies, the present study included pleural fluid collections suspicious for empyema and fluid collections in non-surgical patients into the analysis. This results in a larger patient spectrum but with the same important clinical question, whether a fluid collection is infected or not. Our results can therefore be considered as representative for all fluid collections, which are considered for CT-guided percutaneous drainage. To adjust for better comparability, the subanalyses were performed for postoperative abdominal fluid collections and pleural fluid collections separately. Nonetheless, both analyses showed similar results.

The new proposed score only needs imaging criteria and was more accurate as the score by Radosa et al. and not statistically inferior as the more complex score by Gnannt et al. The new score could be used in clinical routine to guide treatment indication without any need of further anamnestic and/or serological information.

CRP was discussed as the most important clinical parameter to diagnose infected fluid collection [4, 14]. In a clinical case series on surgical patients, the combination of an increased CRP level together with the CT could discriminate patients with a major complication to those without [14]. The proposed threshold was 200 mg/L in this mentioned study is higher compared to the used one of 150 mg/L in the study by Radosa et al. and the threshold of 100 mg/L used by Gnannt et al. The present analysis identified the best accuracy with a threshold of 114 mg/L.

It must be acknowledged that a superior CT based diagnosis of infected fluid collections can reduce possible drainage overtreatment. However, there are also non-infected fluid collections, which might need drainage treatment. For example, non-infected fluid collections with possible mass effect causing symptoms. Yet even for those cases, it could be useful for treatment planning to know the infection state of the fluid collection. For example, choice of drainage type could be influenced by this.

There are several limitations of the present study to address. First, it is a retrospective study with possible known inherent bias. However, imaging and microbiology analyses were performed independently. Second, some serological inflammation parameters, including procalcitonin and Interleukin-6 were not available for all patients, which reflects clinical routine. Moreover, for every patient leucocyte count and CRP were available for analysis, which are most commonly used. Third, possible selection bias can be assumed as we only could include fluid collections undergoing CT-guided drainage treatment. There might be fluid collections not treated by this procedure due to low likelihood for infection or small size without possible drainage placement. Fourth, the imaging was analyzed by one reader. However, it was shown that an excellent interreader agreement can be assumed for the typical findings of infected fluid collections, which reduces possible bias [6]. Fifth, one should consider that preprocessing is important for texture analysis results [15, 16]. There was no complex preprocessing in the present analysis, which could have an influence on the results.

## Conclusions

Albeit texture features derived from CT images were associated with the presence of infection of fluid collections, these were not superior to conventional imaging findings. A score was provided based upon imaging parameters alone, which was comparable to the published scores that are dependent on clinical and serological information. These findings could reduce the need for drainage therapy, which needs to be prospectively evaluated.

## Data Availability

The datasets used and ana- yzed during the current study are available from the corresponding author on reasonable request.

## Abbreviations

CT: Computed tomography
ROI: Region of interest
HU: Hounsfield Unit
CRP: C-reactive protein
